# Systematic review and meta-analysis of bovine cysticercosis in Brazil: current knowledge and way forward

**DOI:** 10.1186/s13071-020-3971-0

**Published:** 2020-02-21

**Authors:** Gabriel Augusto Marques Rossi, Inge Van Damme, Sarah Gabriël

**Affiliations:** 1 Centro Universitário Central Paulista (UNICEP), Rua Miguel Petroni 5111, Postal Code 13563-470 São Carlos, São Paulo Brazil; 2 Department of Veterinary Public Health and Food Safety, Faculty of Veterinary Medicine, Ghent University, Ghent, Belgium

**Keywords:** Beef inspection, Brazil, Bovine cysticercosis, Cattle, Spatial distribution, *Taenia saginata*

## Abstract

**Background:**

*Taenia saginata* taeniosis/cysticercosis has been well studied in several countries. Brazil is one of the most important beef exporting countries and has one of the highest cattle population size in the world. In this country, bovine cysticercosis (BCC) remains the most frequent reported zoonosis detected during *post*-*mortem* inspection, resulting in costs for the beef sector and public health. We performed a systematic literature review regarding data about BCC epidemiology in Brazil and meta-analyses for its prevalence in different administrative regions and the distribution over time, and based on this discussed possible control strategies.

**Methods:**

A systematic review was conducted to obtain data about BCC in Brazil using the words “bovine cysticercosis” and “Brazil” to construct the search phrase. The inclusion criteria used to select articles were: (i) published from 2000 to 2018; (ii) full text available online in Portuguese or English; and (iii) contain information at least regarding one of the following aspects of BCC in Brazil: prevalence, incidence, spatial distribution, risk-factors, economic burden and measures for control.

**Results:**

A set of 42 articles was included, covering the prevalence of BCC in Brazil, ranging between 0.01–18.75%. Prevalence results of 40 articles were included in a meta-analysis per administrative region. The highest prevalence was found in the South (3.4%; 95% CI: 2.0–5.2%), followed by the Southeast (2.7%; 95% CI: 1.9–3.6%), Northeast (1.5%; 95% CI: 0.6–2.7%), Central-western (0.9%; 95% CI: 0.3–1.7%) and North (0.0%; 95% CI: 0.0–0.6%) region. In addition, a reduction in prevalence over time was observed in all the evaluated states except for Alagoas and Pará.

**Conclusions:**

Besides the large availability of data, a critical lack of information about BCC epidemiology remains in Brazil. Nevertheless, the available data on prevalence, high risk-areas and risk factors should contribute to a better understanding of transmission and the formulation of recommendations for control. A One Health approach will be required to reduce *T. saginata* taeniosis/cysticercosis prevalence and the consequent economic burden for the beef sector in Brazil, one of the most important beef exporters in the world.
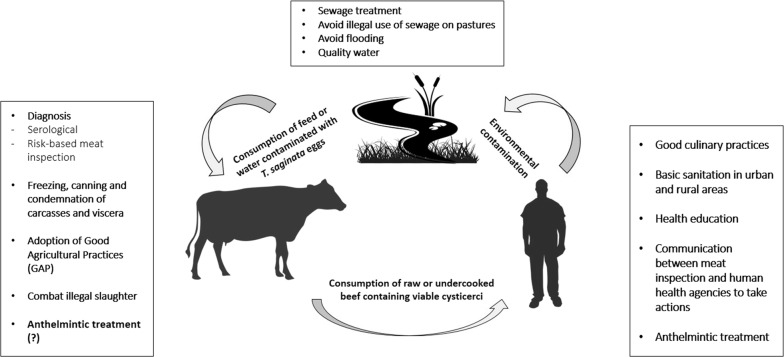

## Background

Bovine cysticercosis (BCC) is an infection caused by the metacestode larval stage of *Taenia saginata* after the accidental ingestion of eggs, mainly through consumption of contaminated feed or water. The environmental contamination originates from the definitive hosts (humans) which harbor the adult tapeworms in their intestines (taeniosis) that release proglottids and eggs daily into the environment, either *via* migrating proglottids or proglottis/eggs in the stool. Taeniosis occurs through consumption of raw or undercooked beef containing viable cysticerci [1], after which an adult tapeworm develops in the intestine, generally without clinical symptoms.

BCC and *T. saginata* taeniosis are widespread in several developing and industrialized countries in Europe [2], Africa [3], the Americas [4], Russian Federation [5] and Asia [6]. For control and prevention of human infections, *post*-*mortem* examinations are performed in cattle slaughterhouses. The latter result in economic burden due to inspection costs, carcass condemnation, costs related to carcass treatment according to national regulations (freezing, drying or canning), the non-export of beef and penalties imposed on farmers supplying cattle infected with cysticercosis [7, 8].

Beef production and export is an important economic activity in Brazil. The cattle population size is estimated at 214.9 million of animals and the country exported 1.64 million of tons of beef in 2018; the highest amount ever exported among all exporting countries [9, 10]. BCC remains endemic in Brazil, with frequent detection during meat inspection resulting in a high economic burden for the beef sector [11]. Considering the very low sensitivity of meat inspection [12], a significant number of viable cysticerci will still reach the consumer through infected beef. From a public health perspective, a relatively high number of taeniosis cases has been described [13, 14], which confirms the food safety issue and requires urgent control [7].

In the last two decades, several researchers have performed studies focused on an improved understanding of the epidemiology and spatial distribution of BCC in order to obtain useful data for the development and adoption of strategies for control. The data obtained in Brazil should contribute to an improved knowledge about BCC prevalence, areas considered with higher risk, risk factors and other variables associated with its occurrence in this country, and the economic burden. Thus, our aims were: (i) to compile and analyze data regarding BCC epidemiology, spatial distribution and economic burden in Brazil; (ii) to perform meta-analyses of BCC prevalence, for different administrative regions and to evaluate the distribution over time; and (iii) based on the obtained data to discuss useful strategies for control.

## Methods

### Study area

**Fig. 1:**
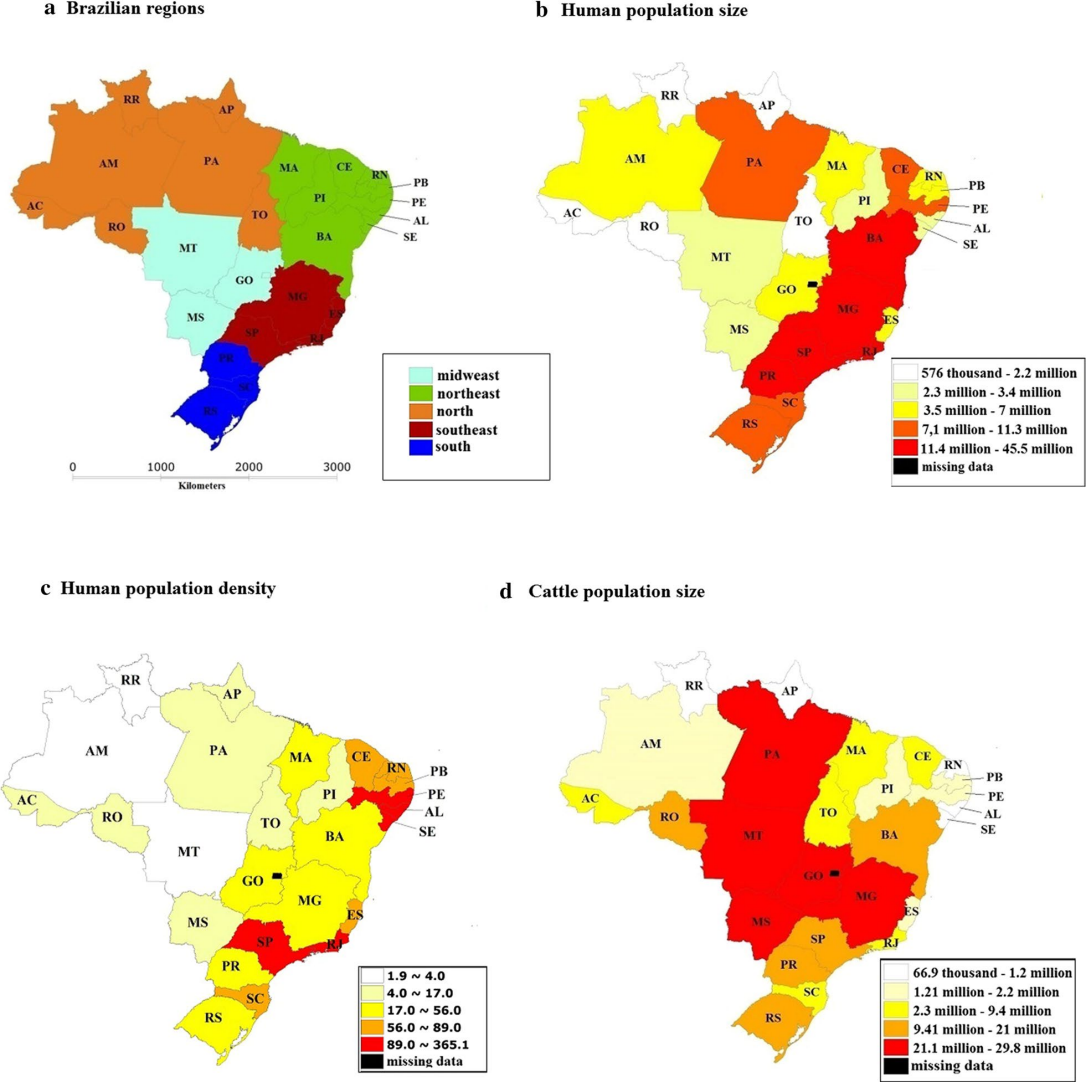
Maps showing administrative regions, human and cattle populational characteristics according to the The Brazilian Institute of Geography and Statistics (IBGE) (https://www.ibge.gov.br/). **a** Brazil is divided into the following states: Acre (AC), Alagoas (AL), Amapá (AP), Amazonas (AM), Bahia (BA), Cerá (CE), Espírito Santo (ES), Goiás (GO), Maranhão (MA), Mato Grosso (MT), Mato Grosso do Sul (MS), Minas Gerais (MG), Pará (PA), Paraíba (PB), Paraná (PR), Pernambuco (PE), Piauí (PI), Rio de Janeiro (RJ), Rio Grande do Norte (RN), Rio Grande do Sul (RS), Rondônia (RO), Roraima (RR), Santa Catarina (SC), São Paulo (SP), Sergipe (SE) and Tocantins (TO), which are divided into five Brazilian regions (Midwest, Northeast, North, Southeast and South). **b** Human population size estimated for 2018 in 26 states. **c** Human population density estimated for 2018 in 26 states. **d** Cattle population size in 2017. The maps were created in Terraview® Software (INPE, São José dos Campos, Brazil, v.4.2.2) (http://www.dpi.inpe.br/terraview)

Brazil is the largest country in South America (area of 8 million km^2^) and has over 208 million inhabitants distributed over 5570 municipalities. The Federative Republic of Brazil is composed of the union of 27 federative units: 26 states and 1 Federal District (DF) (located in Goiás State) (Fig. 1).

### Search strategy

We followed the PRISMA guidelines for systematic reviews [15] (Additional file 1: Table S1). A review of literature published from 2000 to 2018 was conducted to obtain data about the prevalence, geographical distribution, risk factors and other variables associated with BCC, its economic burden and recommendations for BCC control in international bibliographic databases included in Google Scholar (https://scholar.google.com.br/). As our focus was strictly on BCC and not taeniosis, the keywords (“Bovine cysticercosis” AND “Brazil”) OR (“Cisticercose bovina” AND “Brasil”) (Portuguese), were used to construct the search phrase in this database. The specific time interval was constructed as 2000 (initial) and 2018 (final).

**Fig. 2:**
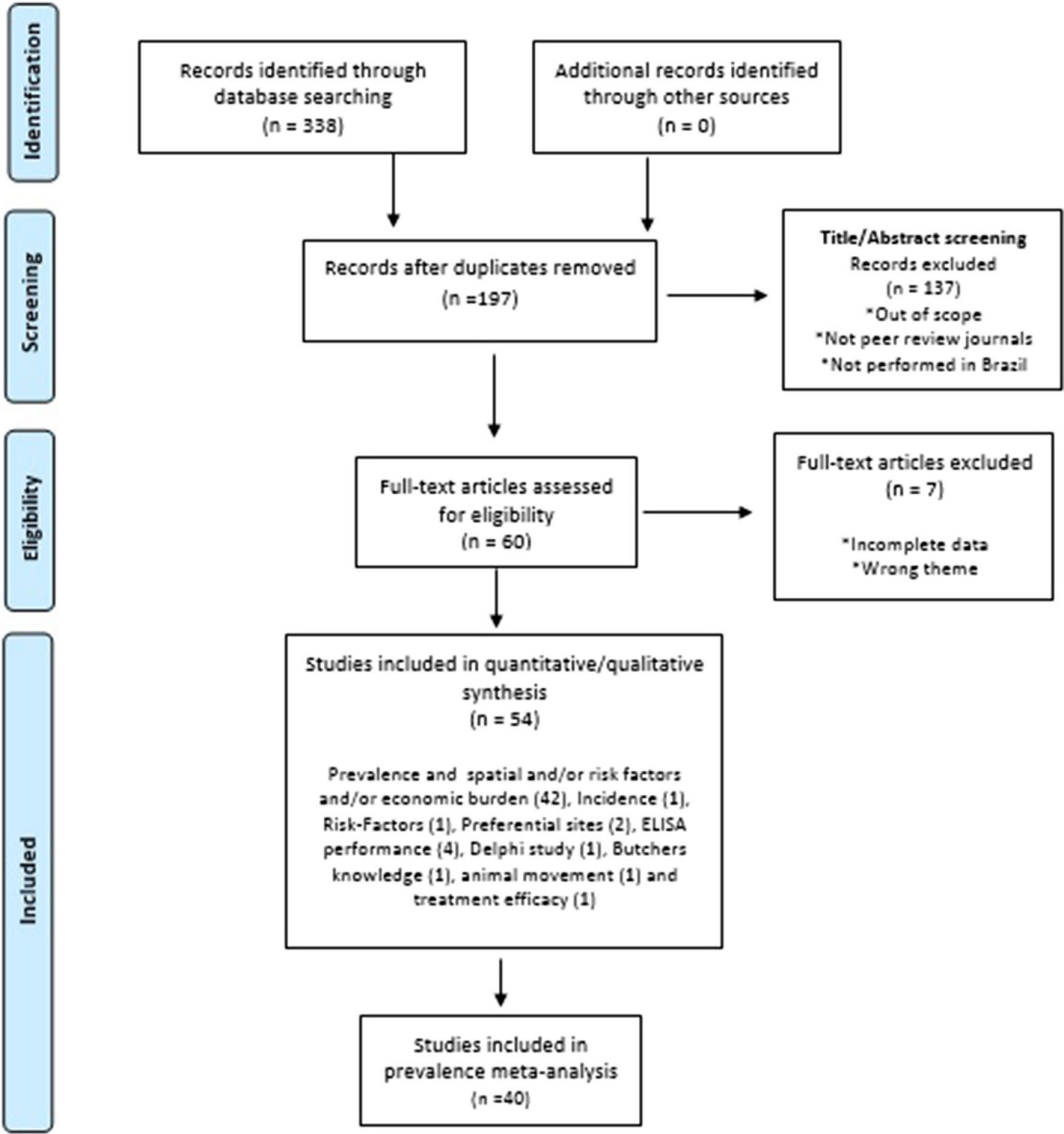
Prisma flowchart diagram of the record selection process

Subsequently, the compilation was performed, the duplicate records were removed and the relevance of the results was analyzed. The following inclusion criteria were used to select articles: (i) studies performed in Brazil; (ii) published in peer review journals from 2010 to 2018; (iii) full text available online in Portuguese or English; and (iv) contain information at least regarding one of these aspects of BCC in Brazil: prevalence, incidence, spatial distribution, risk-factors, economic burden and measures for control (Fig. 2). The articles considered as not eligible were those published before 2000 or after 2018 and/or with no access to full text and/or not performed in Brazil and/or out of scope.

### Data analysis

Quantitative data were stored in a predefined spread sheet document, including the study area (state), period of the study, authors and year of publication, number of animals, number of infected animals, prevalence, method of detection and economic burden (when available). Additionally, data were recorded per state, so for studies that reported BCC data in different states, different rows (one for each state) were created. Another spreadsheet was used to store data about risk-factors studies. Qualitative data on high-risk areas or related to BCC control were extracted and compiled in other tables.

All analyses were carried out using R version 3.5.2 [16]. A meta-analysis was performed on the occurrence of BCC in Brazil according to Wang [17] using the *meta* package (version 4.9-6) [18]. The double arcsine transformation was applied for proportions (number of cases/total number of animals) prior to analysis. A subgroup analysis was performed to calculate a prevalence estimate per administrative region, assuming a common between-study variance. For studies reporting results for multiple states, data of different states within the same region were combined by summing the number of positive cases and the total number of animals.

To evaluate the effect of time on the occurrence of BCC in Brazil, results were recorded per state/year combination. For state/year combinations that were reported by multiple studies, the numbers of different studies were combined by adding the number of positive cases and the total number of animals tested. A logistic regression was used to evaluate the effect of time on the occurrence of BCC, using the year (as a continuous variable) and state (as a factor variable) as main effects and including the two-way interaction between year and state to allow for a different time effect in each state. To evaluate if the interaction term was significant, a likelihood ratio (LR) chi-square test was used.

## Results

A set of 42 peer-reviewed published articles containing prevalence values (Additional file 2: Table S2) [7, 11, 19–58] and one describing the incidence of BCC in areas in Brazil was found in literature [59]. From these articles, we identified two articles [11, 26] describing BCC prevalence in several Brazilian states, covering a long period and including a large sample size. In addition, we found 12 articles containing information about the spatial distribution of BCC inside/within the states (Table 1), 13 articles describing risk factors or variables associated with BCC occurrence (Table 2) and 4 describing the economic burden of BCC [7, 22, 28, 30]. Furthermore, a set of 10 studies performed in Brazil were also included, regarding at least one aspect presented in discussion section, such as efficacy of chemical treatment (*n* = 1) [62], preferential infection sites (*n* = 2) [63, 64], development of serological tests (*n* = 4) [65–68] and measures for BCC control (*n* = 3) [69–71].

**Table 1:** Bovine cysticercosis high-risk areas within nine Brazilian states

State	Method	Areas with higher risk	
Bahia	PM inspection	101 municipalities located at Itapetinga, Litoral Sul, Médio Rio de Contas, Vitória da Conquista and Extremo Sul territories	Bavia et al. [33]
Espírito Santo	PM inspection	Counties: Ecoporanga, Linhares, Presidente Kennedy and Itapemirim	Avelar et al. [28]
Goiás	PM inspection	The Central mesoregion was considered as the one with the highest prevalence and the microregions of Goiânia, Anápolis, Pires do Rio, Vale do Rio dos Bois, Meia Ponte e Anicuns (OR > 5)	Aquino et al. [22]
São Paulo	PM inspection	Highest prevalence in regions Central, Ribeirão Preto and Presidente Prudente; higher probability of finding infected animals in regions of Araçatuba, Barretos, Bauru, Franca and Sorocaba	Ferreira et al. [39]
	PM inspection	Higher risk in the administrative regions São José do Rio Preto and Campinas	Rossi et al. [25]
Paraná	PM inspection	Municipalities of Campo Largo, Capanema, Rosário do Ivaí, Japira, Joaquim Távora, Laranjeiras do Sul, Rio Bonito do Iguaçu, Palmas, Saudades do Iguaçu and Antônio Olinto	Souza et al. [45]
	PM inspection	Higher prevalence in nucleus of Curitiba, Francisco Beltrão and Irati; higher OR in nucleus of União da Vitória, Francisco Beltrão and Irati	Guimarães-Peixoto et al. [29]
Mato Grosso	PM inspection	Highest OR in the administrative regions Sinop, Barra do Garças, Água Boa, Cáceres, Barra do Bugres, Cuiabá, Pontes Lacerda, Rondonópolis, Matupa, São Félix do Araguaia and Lucas do Rio Verde	Rossi et al. [21]
Mato Grosso do Sul	PM inspection	Higher risk in the administrative regions Amambai, Navirai, Nova Andradina, Dourados, Três Lagoas, Campo Grande, Ponta Porã, Costa Rica, Aquidauana and Coxim	Pereira et al. [23]
	PM inspection	Municipalities of Dourados and Santa Rita do Rio Pardo	Concenço et al. [60]
Paraíba	ELISA and immunoblot	Higher prevalence in animals in Borborema, Agreste/Zona da Mata and Sertão	Maia et al. [27]
Rondônia	PM inspection	Higher risk in the administrative regions Porto Velho, Guajará-Mirim, Colorado D’Oeste, Cacoal, Ji-Paraná	Alves et al. [24]

**Table 2:** Variables associated with risk factors for bovine cysticercosis according to 13 studies performed in Brazil

Variable	Area	Methodology	Reference
Raising animals in regions where coffee, orange and sugarcane are harvested	São Paulo State	Cluster analysis of selected variables and prevalence in municipalities based on *post-mortem* data	Rossi et al. [25]
Access of cattle to non-controlled water sources and sport fishing activities near the farms	States of São Paulo, Minas Gerais, Mato Grosso and Mato Grosso do Sul	Case–control study in farms based on *post-mortem* data	Rossi et al. [7]
Animal purchasing and presence of flooded pastures	Paraíba State	Logistic regression of results obtained for herd-level using serological analyses	Maia et al. [27]
Raising animals in regions with large human population	Mato Grosso State	Logistic regression of selected variables and prevalence in municipalities based on *post-mortem* data	Rossi et al. [21]
Ingestion of undercooked beef by humans and occurrence bovine cysticercosis in animals	São João do Evangelista, Minas Gerais	Association analysis between results from cattle serological analyses and questionnaire completed by humans in sampled farms	Garro et al. [41]
	Salinas, Minas Gerais		Magalhães et al. [48]
Expertise of those responsible for the farm, the family income and water quality	Triangulo Mineiro, Minas Gerais	Logistic regression of results from cattle serological analyses and questionnaire completed by humans in sampled farms	Duarte et al. [37]
Raising animals in areas with high educational human development index or where sugarcane and coffee are harvested	São Paulo State	Maps	Ferreira et al. [39]
Raising animals in regions with large human population and rainfall index (positive correlation) and large size of cattle population in municipalities (negative correlation)	Mato Grosso do Sul State	Correlation analysis of selected variables and prevalence in municipalities based on *post-mortem* data	Pereira et al. [23]
Bovine meat for human consumption acquired in the city and farm	Viçosa country, Minas Gerais	Logistic regression of results from cattle serological analyses and questionnaire completed by humans in sampled farms	Santos et al. [43]
Farm size greater than 301 hectares	Colatina, Espírito Santo	Case–control study in farms	Acevedo-Nieto et al. [61]
Interference of the rivers and their tributaries that fed the municipalities	Triangulo Mineiro, Minas Gerais,	Risk analysis along with mapping and spatial analysis of data	Duarte et al. [47]
Raising animals in regions with large human population, percentage or urban houses and rural areas with inappropriate sewage system	Rondônia	Correlation analysis of selected variables and prevalence in municipalities based on *post-mortem* data	Alves et al. [24]

### Incidence, prevalence and spatial distribution in Brazil

There was only one study performed in Brazil which describes the incidence of BCC from 2013 to 2016 in five Brazilian municipalities located in the state of Rio Grande do Sul (RS): Arroio Grande (0.72%), Canguçu (0.58%), Capão do Leão (1.31%), Pelotas (1.06%) and São Lourenço (0.83%) [59]. Furthermore, a set of 42 articles was found containing prevalence values and other additional information such as the period, method, state, administrative region, number of examined animals and number of cases (Additional file 2: Table S2).

*Post-mortem* inspection (meat inspection) was the most used method (34 articles) to detect infected animals, with only eight studies using serological tests (ELISA as trial and immunoblot confirmatory). The BCC prevalence described in these 42 studies ranged from 0.01% in the state of Rondônia [34] to 18.75% in indigenous villages in the state of Mato Grosso do Sul [35].

**Fig. 3:**
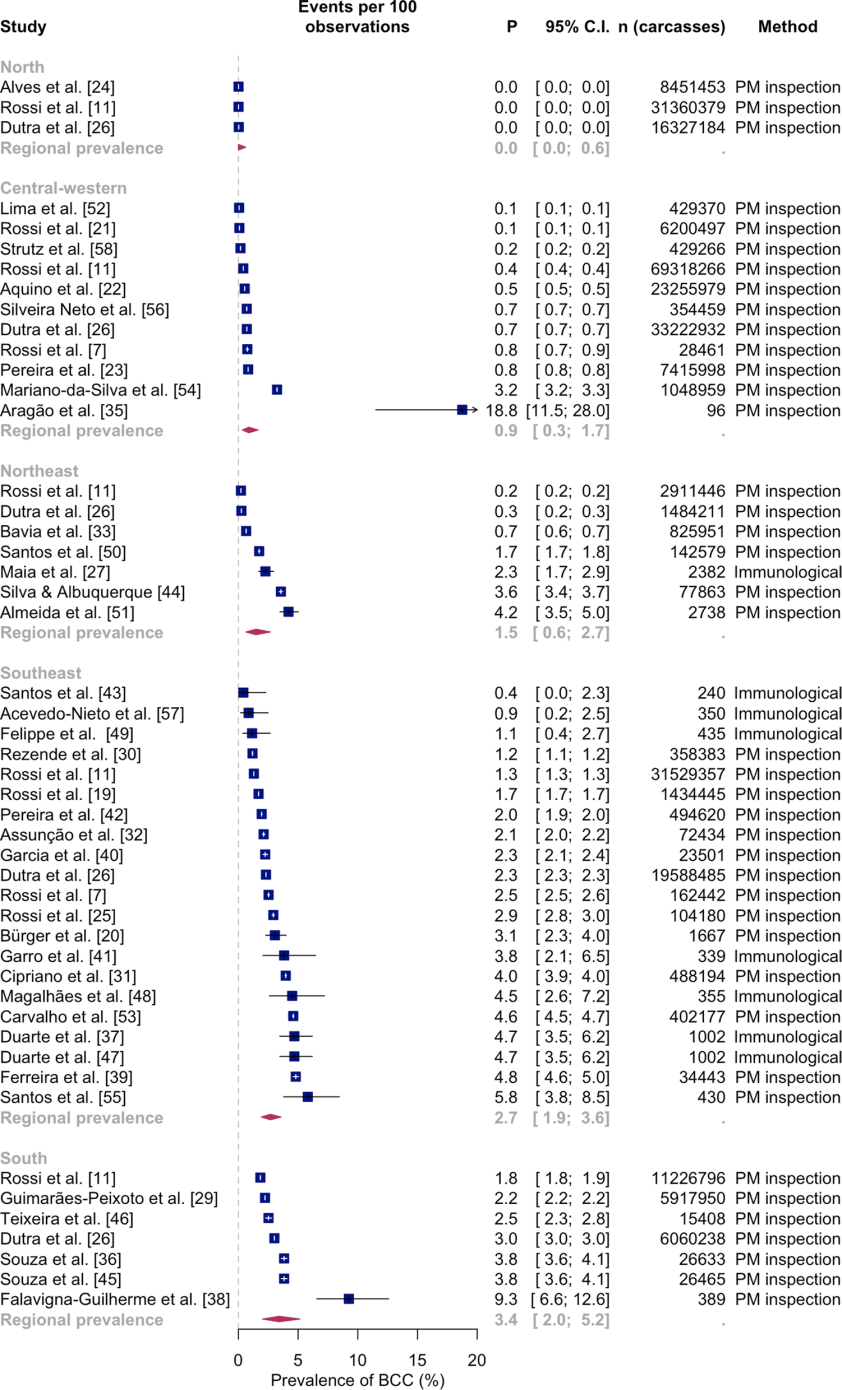
Forest tree of 40 studies reporting BCC prevalence in Brazil, grouped per administrative region (North, Northeast, Central-Western, Southeast and South)

Forty studies were included in the meta-analysis of BCC in the different Brazilian regions (Fig. 3). One study was excluded because the total number of animals was not provided [28] and another study was excluded as animals from three states (SP, MG and GO) were used without specifying the number of animals per state [34]. Most studies were conducted in the Southeast region (*n* = 21), whereas only three studies examined BCC in the North region. The highest prevalence was found in the South region (3.4%; 95% CI: 2.0–5.2%), followed by the Southeast (2.7%; 95% CI: 1.9–3.6%), Northeast (1.5%; 95% CI: 0.6–2.7%), Central-western (0.9%; 95% CI: 0.3–1.7%) and North regions (0.0%; 95% CI 0.0–0.6%) (Fig. 3).

Only two studies using *post-mortem* inspection [11, 26] included animals from several Brazilian states and sampled a high number of animals (75,983,590 and 146,346,244 animals, respectively) covering a long period (more than three years). Dutra et al. [26] included animals from Acre, Alagoas, Amazonas, Bahia, Espírito Santo, Goiás, Mato Grosso, Mato Grosso do Sul, Minas Gerais, Pará, Paraná, Rio de Janeiro, Rio Grande do Sul, Rondônia, Santa Catarina, São Paulo, Sergipe and Tocantins from 2007 to April 2010, while Rossi et al. [11] studied the period from 2010 to 2015 using animals from the same states except for Alagoas. These studies were used to evaluate the occurrence of BCC over time.

**Fig. 4:**
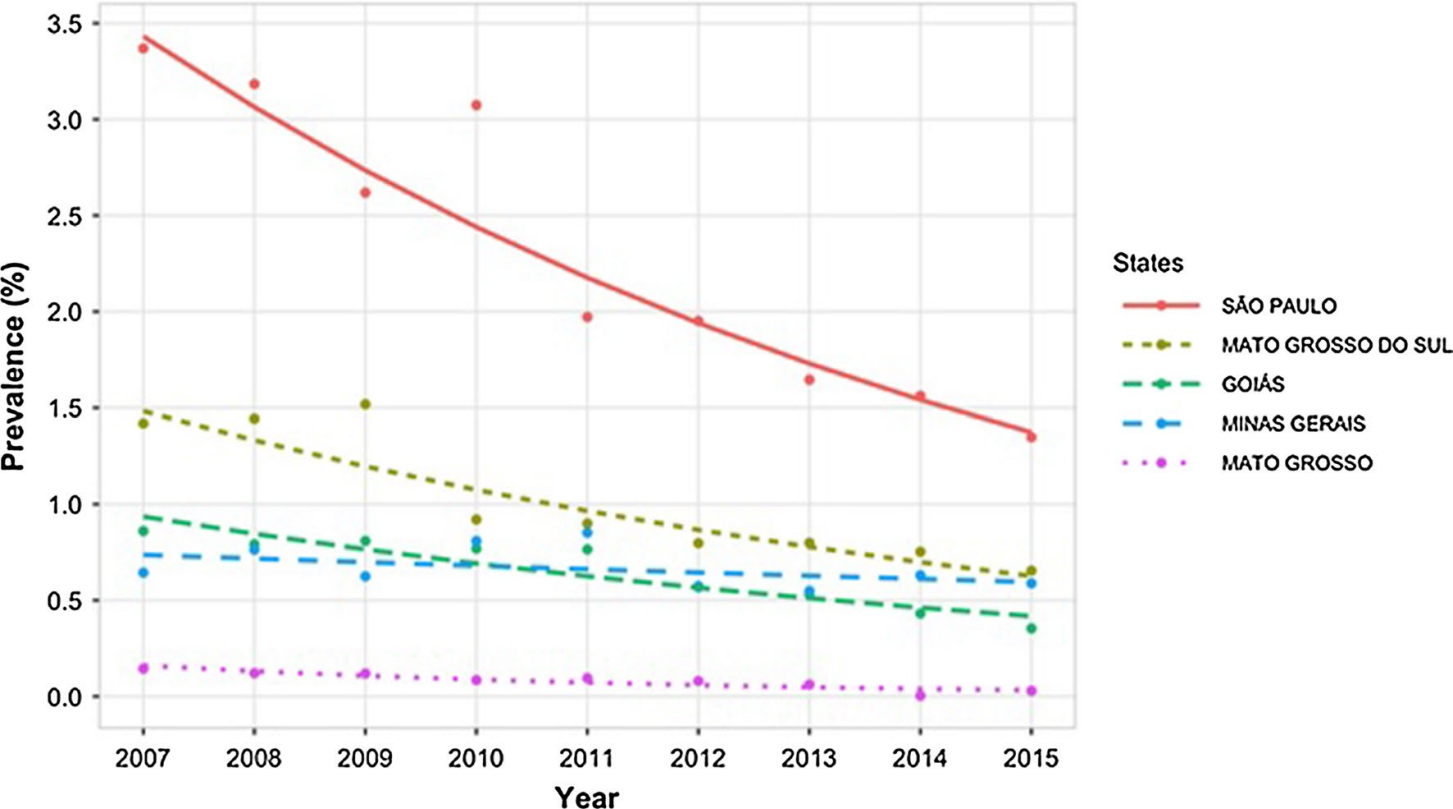
Time distribution of BCC prevalence in five Brazilian states where most data regarding BCC were available (Goiás, Mato Grosso, Mato Grosso do Sul, Minas Gerais and São Paulo) from 2007 to 2015. The points represent the observed data and the lines are the predicted probabilities. Data are from Dutra et al. [26] and Rossi et al. [11]

The prevalence of BCC significantly decreased over time (LR *χ*^2^ = 125044, *df*  = 1, *P* < 0.001), although the time effect differed between the different states (LR *χ*^2^ = 9029, *df*  = 17, *P* < 0.001). A reduction in BCC prevalence was observed for all included states, except for Pará and Alagoas. The observed and predicted time distribution of BCC in the five states with most data (Goiás, Mato Grosso, Mato Grosso do Sul, Minas Gerais and São Paulo) is illustrated in Fig. 4. The states that had the highest prevalence of bovine cysticercosis were Rio Grande do Sul, Santa Catarina, São Paulo and Paraná. The observed prevalence within these states varied between 2.8–3.7% in 2007 [26]. Although the prevalence in these five states decreased to 1.3–1.5% in 2015 [11], they remained the highest among the different states. In Pará, Alagoas, Amazonas and Tocantins, the prevalence remained below 0.5% throughout the entire study period (2007–2015).

In addition, the spatial distribution within nine Brazilian states also has been studied (Table 1). These data summarize the areas considered with a higher risk or prevalence of BCC within nine states using data from 12 studies.

### Risk factors

There were several variables considered as risk factors for BCC in Brazil, which are presented in the 13 studies included in Table 2. Briefly, these factors were linked to areas with large human population, temporary workers involved in crop production, cattle access to uncontrolled water sources, animal purchasing, failures in sanitary education, basic sanitation, rainfall index and farm and farmers characteristics.

### Economic burden

BCC leading to reduced carcass value or total carcass condemnation at slaughter, results in important economic losses for the Brazilian beef sector. Four articles presented data about the economic burden [7, 22, 29, 30].

A total of 29,708,550 kg of beef was condemned for cysticercosis during 2004–2008 in Paraná State, resulting in an economic burden of around USD 31,915,700 due to carcass condemnation [29]. The economic burden for farmers was reported for other states as well, such as: (i) in Minas Gerais State, where farmers had economic losses of USD 537,526.80 due to the detection of 4243 infected bovines from 2009 to 2016 [30]; (ii) states of Minas Gerais, São Paulo, Mato Grosso and Mato Grosso do Sul, where a group of farmers delivering animals to a single slaughterhouse lost USD 312,194.52 during one year [7]; and (iii) in Goiás State, where farmers had economic losses ranging from USD 9,260,728.57 to 11,313,816.67 from 2007 to 2014 [22].

## Discussion

### Epidemiology

High human population density has been reported as a risk factor for BCC in Brazil [21, 23]. In some Brazilian states, such as São Paulo, Espírito Santo, Minas Gerais, Paraná, Santa Catarina, Bahia, Paraíba and Rio Grande do Sul, a high prevalence was observed (mostly > 2%) (Additional file 2: Table S2). These states are located mainly in the Southeast and South regions, which had the highest BCC prevalence values in the prevalence meta-analyses and also a higher human population density (86.82 and 48.58 inhabitants/km^2^, respectively) [72]. In the UK, farms situated close to a permanent potential source of human fecal contamination are considered with a higher risk for BCC [73] as the presence of infected humans results in environmental contamination with *T. saginata* eggs, mainly in areas with inappropriate sewage systems [24]. The wastewater treatment in Brazil evolved from 42% of the Brazilian human population in 2007 to 50.3% in 2015, leaving more than 100 million of inhabitants without proper sanitation. According to the Brazilian Institute of Geography and Statistics, only 55.16% of the 5565 Brazilian municipalities had sewerage systems in 2008. There is a difference in the percentage of municipalities containing sewage collector systems among Brazilian regions. The South region is the one with the highest value (95.08% of the municipalities) followed by the Northeast (45.68%), South (39.73), Central-western (28.33%) and North (13.36%) regions [74]. However, the Southeast is the second region with a high prevalence of BCC, demonstrating that basic sanitation is not enough to avoid animal’s infection through the ingestion of *T. saginata* eggs. Open defecation and underuse of sanitary facilities have also been demonstrated to contribute to maintain taeniosis/cysticercosis in endemic areas [75] and these practices could contribute to BCC transmission in Brazil but data are lacking to assess the magnitude.

In Brazil, beef cattle are raised mostly extensive [76], where cattle feeding occurs in large areas of pastures with free access to uncontrolled water sources. The relation with access to contaminated water has been described by several authors in Brazil, detailing risks such as the free access of cattle to uncontrolled water sources [7], the presence of flooded pastures [27] or areas with a high rainfall index [23], proximity to rivers and their tributaries that fed municipalities [47], and poor quality of water [37]. Similar risk factors have been reported in other countries, such as the access to risky water sources with sewage treatment effluent plant in proximity [77], the flooding of pastures and free access to surface water, and the proximity of wastewater effluent [78]. Water supply for animals appears to be the most frequent route of infection for animals in Spain [79].

Raising animals near areas where sugarcane, coffee and orange are harvested has been identified as a risk factor for BCC in Brazil, which is probably due to the presence of temporary workers [25, 39]. Similarly, hiring contractors has been considered as a risk factor for BCC in Denmark [77, 80]. In Brazil, the adoption of new technologies in crop production, leading to a decrease in human labor needs, could, in combination with the increase of proper sewage treatment systems, explain the BCC reduction over the time (Fig. 4).

Other important factors related to BCC have been shown in other countries and may be applicable to Brazil. Allowing animals outdoor access (grazing) is a risk factor reported from Denmark [77, 80] and a common practice in Brazil, where animals have free access to large pastures [76]. Brazil has a large dairy production chain producing 33.8 billion liters of raw milk during 2018 [81], whereby the old dairy cows, may be at a higher risk as reported in Denmark [80] and Spain [79]. Large farms with high numbers of animals are other reported risk factors [78, 79] that may apply to the Brazilian conditions.

### Economic burden

Globally, only few countries have made in depth calculations on the economic impact of *T. saginata*. In northeastern Spain, where the prevalence is low (0.010% from 2008–205), the overall impact of *T. saginata* amounted to €154,903/year during 2013 to 2015 and meat inspection accounted for 81.9% of the costs, followed by carcass condemnation and freezing (9.4%) and taeniosis (8.7%) [82]. In Belgium, the highest proportion of the total economic losses is borne by cattle farmers (economic cost of €3,408,455/year), mainly due to insurance fees. Cost related to taeniosis amount to €795,858/year [8]. Both studies highlight the lack of available data that would allow more accurate assessments. The same is true for Brazil, for which only four articles were found that evaluated the economic burden of BCC based on condemnation in slaughterhouses, varying between USD 312,194.52 (for a group of farmers at the states of Minas Gerais, São Paulo, Mato Grosso and Mato Grosso do Sul, which supplied a single slaughterhouse during 2012) and USD 31,915,700 (in Paraná between 2004 and 2008). Beef production is an important industry in Brazil, with a Livestock Gross Domestic Product (GDP) in 2018 around USD 144 billion, being responsible for 8.7% of the Brazilian GDP [83]. While the reported articles provide important information, results are fragmented and do not allow establishment of the real economic impact, requiring further studies for a better comprehension.

### How to control *Taenia saginata* in Brazil?

**Fig. 5:**
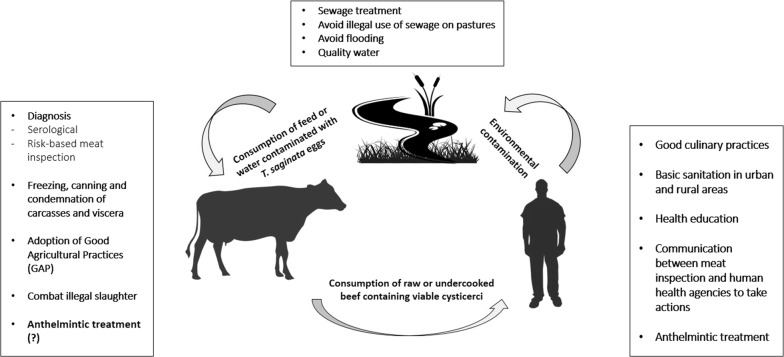
Recommended measures for *T. saginata* cysticercosis/taeniosis control

For the control of parasitic zoonosis, such as taeniosis, a “One Health” approach including human, animal and environmental health have been suggested [84, 85]. To achieve sustained control, a multidisciplinary approach should be implemented (Fig. 5), joined with a monitoring and surveillance programme.

The most commonly applied control strategy is the detection of infected carcasses at slaughter *via* visual meat inspection [86]. The *post-mortem* examination of carcasses aims to avoid infected animals entering into the food supply chain. However, meat inspection is notorious for its low sensitivity, mainly in carcasses with light infections [87–89] which are common in Brazil [7]. According to some studies, the sensitivity of meat inspection ranged from 0.54% [12] up to 2.87% in an enhanced meat inspection system [90] in other countries. The *post-mortem* inspection for BCC is performed through visual inspection and multiple incisions in muscles (masseters, pterygoids, tongue and heart). If cisticerci are detected, the lesions are identified and the half carcasses, together with the viscera and the head, are sent to the Final Inspection Department (DIF), where they are examined by a veterinarian [91, 92] through complementary visual examination, palpation and incisions in the head, liver, esophagus, diaphragm and the carcass’s external surfaces. According to Brazilian literature, the detection occurs mainly during exams of head muscles, liver, tongue and heart [45], but the parasite can be found in other sites such as chuck, rump, strip loin, full tenderloin, back ribs and shoulder [63, 64]. The Brazilian Regulation of Industrial and Sanitary Inspection of Animal Products (RIISPOA) states that animals with heavy infections must be condemned. According to Brazilian law, heavy infections are characterized using the following criteria: at least eight cysticerci (viable or not viable) distributed as follows: (i) two or more cysticerci simultaneously in two preferential sites (masseter and pterygoid muscles, tongue, heart, diaphragm, liver and esophagus), totalizing four cysticerci; and (ii) four or more cysticerci on the chuck, brisket and shank, or on loins and round [92]. Carcasses with a single viable cysticercus must have it removed and be heat-treated using − 10 °C during at least ten days or through salt use during 21 days, while carcasses containing only one not viable cysticercus must have it removed, also, the carcass is considered unsuitable for export. Finally, moderate infections (more than one cysticercus, but lower than the heavy infections) require canning or cooking at a temperature of 76.6 °C for at least 30 min [92]. No studies have been performed assessing the sensitivity of the Brazilian meat inspection system.

Some authors have been suggesting to perform meat inspection on animals categorized according to their risk of harboring cysticerci, because it is thought more efficient and sensitive than traditional methods [93, 94]. This risk-based system could be assessed for Brazil, as there are several risk factors and associated variables described for BCC in this country (Table 2). The use of serological analyses, such as Ag-ELISA, to detect infected animals also has been suggested but might not be feasible during slaughter [12]. However, serological analyses have been largely carried out, including in Brazil [65–68] and its use to detect infected animals has improved worldwide [90, 95, 96], allowing to perform well-designed epidemiological studies.

Considering the low sensitivity of meat inspection and problems related to the detection of infected carcasses using other methods, other strategies for BCC control are required in Brazil, in order to interrupt taeniosis/cysticercosis transmission. According to a Delphy study performed by experts in BCC epidemiology, there are six categories of control measures: (i) health education; (ii) health intersectorality; (iii) health surveillance and legislation; (iv) sanitation measures; (v) epidemiological studies; and (vi) methods of diagnosis and treatment [70].

Johansen et al. [97] highlighted that “ignorance is the major obstacle for the effective control of diseases”; indeed, educating the population about amongst others sanitation and the consumption of well-cooked beef is an important strategy to interrupt *T. saginata* taeniosis/cysticercosis transmission [70]. Children are recognized as excellent health change agents [98, 99], highlighting the need to include them in educational programmes. A study including middle and high school students in Brazil showed that approximately 75% of interviewed students had never heard the words “cysticercosis” or “taeniosis” [20]. Besides education of the general population, targeted information for specific groups such as farmers, butchers, should be addressed as well, as the ignorance about cysticercosis/taeniosis was also shown in butchers in Brazil [69]. Electronic educational tools have been successfully used for *T. solium* control in endemic countries [97, 100, 101] and the adoption of a similar tool for *T. saginata* could be useful in Brazil. Specific flyers and information notes have been developed in the framework of CYSTINET, the European Network for taeniosis/cysticercosis (COST Action TD1302) (http://www.cystinet.org), which may also be adapted to the Brazilian situation.

To date, beef inspection services only communicate with the animal’s owner when cysticercosis is detected during slaughter. Unfortunately, this is not followed by an investigation or activity conducted by animal health agencies. The detection of cysticercosis during *post-mortem* exams should be communicated to health departments, including origin and farm location, so that actions, i.e. including health education programmes and human treatment, may be taken on the different levels including integrated activities among professionals of the program for family health, educators and workers [70].

The identification of the locations where the animals become infected is often complicated in Brazil, due to the movement of animals between farms in the course of their lives. Animal’s movement can complicate the interpretation of results obtained from epidemiological studies [102]. The use of animal movement network analysis to map farms serving as contamination sources have been studied in Brazil with interesting results. The detection of farms with risk of *T. saginata* infection using this network along with the proper sanitary management and human treatment resulted in a decrease in BCC prevalence, from 25% in 2010 to 1.8% in 2012 [71].

Another control measure that needs improvement in Brazil is the combat against illegal slaughter [70], a recognized practice that occurs in Brazil, allowing infected animals to enter in food supply chain. There are no official data for the current situation of illegal slaughter in Brazil.

Anthelmintic treatment of infected cattle has been suggested; however, the currently available and tested anthelmintics (albendazole sulphoxide and albendazole) gave inconsistent efficacy results [62]. Since animal treatment in Brazil has been rather unsuccessful, a better health management for cattle remains key in controlling *T. saginata* [70]. It means the adoption of practices able to interrupt the transmission of BCC through ingestion of eggs present in contaminated water, pastures and animal food, such as basic sanitation or proper animal management. As previously discussed, there is a risk for grazing in contaminated pastures due overflooding of rivers contaminated with *T. saginata* eggs or drinking contaminated water. The adoption of good agricultural practices (GAP) in beef farms including measures such as to avoid the ingestion of uncontrolled water sources or contaminated food must contribute to its control. The slaughterhouses must encourage and require this quality tool from farms during implementation of hazard analysis and critical control points (HACCP) in order to avoid risk for consumers due consumption of viable cysticerci in beef [7].

## Conclusions

Besides the large amount of data available about the occurrence and risk factors of cysticercosis in Brazil, which contributed to improve the knowledge, a critical lack of information still remains, mainly regarding the economic impact and assessments of strategies for BCC control. There is an urgent need for interventions through a “One Health” approach in order to continue reducing the BCC prevalence in Brazil, contributing to improving human health and reducing the economic burden for the beef sector in one of the most important beef-exporting countries in the world.

## Supplementary information

**Additional file 1: Table S1.** PRISMA checklist.

**Additional file 2: Table S2.** The set of 42 studies performed in areas located at Brazil published from 2000 to 2018 presenting the period, method, state, administrative region, number of examined animals, number of cases and prevalence of BCC in Brazil.

## Data Availability

The data supporting the conclusions of this study are provided within the article. All datasets generated and analyzed during this study are available from the corresponding author upon reasonable request.
